# The hypoxia-related signature predicts prognosis, pyroptosis and drug sensitivity of osteosarcoma

**DOI:** 10.3389/fcell.2022.814722

**Published:** 2022-09-20

**Authors:** Lin Hu, Xin Wu, Dongjie Chen, Zhenyu Cao, Zian Li, Yanmin Liu, Qiangqiang Zhao

**Affiliations:** ^1^ Department of Pediatrics, The Third Xiangya Hospital, Central South University, Changsha, China; ^2^ Department of Spine Surgery, Third Xiangya Hospital, Central South University, Changsha, China; ^3^ Department of Hepatopancreatobiliary Surgery, The Third Xiangya Hospital, Central South University, Changsha, China; ^4^ Department of Orthopedics, The Qinghai Provincial People's Hospital, Xining, China; ^5^ Department of Clinical Laboratory, Qinghai Provincial People's Hospital, Xining, China; ^6^ Department of Cardiovascular Medicine, The Qinghai Provincial People's Hospital, Xining, China; ^7^ Department of Hematology, The Qinghai Provincial People’s Hospital, Xining, China

**Keywords:** hypoxia, osteosarcoma, pyroptosis, drug resistance, prognosis

## Abstract

Osteosarcoma (OS) is one of the most common types of solid sarcoma with a poor prognosis. Solid tumors are often exposed to hypoxic conditions, while hypoxia is regarded as a driving force in tumor recurrence, metastasis, progression, low chemosensitivity and poor prognosis. Pytoptosis is a gasdermin-mediated inflammatory cell death that plays an essential role in host defense against tumorigenesis. However, few studies have reported relationships among hypoxia, pyroptosis, tumor immune microenvironment, chemosensitivity, and prognosis in OS. In this study, gene and clinical data from Therapeutically Applicable Research to Generate Effective Treatments (TARGET) and Gene Expression Omnibus (GEO) databases were merged to develop a hypoxia risk model comprising four genes (*PDK1, LOX, DCN,* and *HMOX1*). The high hypoxia risk group had a poor prognosis and immunosuppressive status. Meanwhile, the infiltration of CD8^+^ T cells, activated memory CD4^+^ T cells, and related chemokines and genes were associated with clinical survival outcomes or chemosensitivity, the possible crucial driving forces of the OS hypoxia immune microenvironment that affect the development of pyroptosis. We established a pyroptosis risk model based on 14 pyroptosis-related genes to independently predict not only the prognosis but also the chemotherapy sensitivities. By exploring the various connections between the hypoxic immune microenvironment and pyroptosis, this study indicates that hypoxia could influence tumor immune microenvironment (TIM) remodeling and promote pyroptosis leading to poor prognosis and low chemosensitivity.

## Introduction

Osteosarcoma (OS), currently the most common primary solid bone malignancy, is derived from primitive mesenchymal cells and usually occurs in children and young adults (Li et al., 2011; Smeland et al., 2019). The incidence of OS is over 8 million/year at 15–19 years of age in Europe (Ritter and Bielack, 2010). OS is severely malignant; a considerable number of patients with the seemingly localized disease will develop metastases mostly in the lungs (Goguet-Surmenian et al., 2013). Thus, a multidisciplinary treatment approach is very important. To date, the 5-year survival of OS has improved and is achieved by almost 70% of the patients via the advancement of neoadjuvant and adjuvant chemotherapy combined with surgical resection (Rosen, 1985; Lilienthal and Herold, 2020). However, OS is a chemotherapy-resistant solid tumor with many patients being insensitive to chemotherapy due to the highly dynamic immune landscape in patients with OS ((Wu et al., 2021a), (Wu et al., 2021b)). Thus, a deep understanding of the tumor immune status in OS is crucial for improving prognosis and selecting effective treatments.

Hypoxia plays an important role in solid tumor development including OS. Specifically, hypoxia is a significant factor that affects tumor cell proliferation and invasion (Guan et al., 2015; Chen et al., 2020). The imbalance between oxygen consumption and supply results in hypoxia, an inevitable phenomenon in the majority of tumors (Zeng et al., 2011). Hypoxia is a relevant factor in chemosensitivity, metastatic potential, malignant phenotype, and poor survival (Song et al., 2006; Guan et al., 2015; Prudowsky and Yustein, 2020). Several studies have also found that the complicated relationship between hypoxia and the tumor microenvironment has been shown to occur in various tumors such as hepatocellular carcinoma, glioma, gastric cancer, acute myeloid leukemia, and breast cancer (Gibadulinova et al., 2020; Lin et al., 2020; Liu et al., 2020; Du et al., 2021; Zeng et al., 2021; Jiang et al., 2022). Hypoxia can decrease immune cell (e.g., CD8^+^ T cells) proliferation and promote disadvantageous expression of immune factors (e.g., HMGB1 and TLR4) (Yang et al., 2014; Liu et al., 2015; Wu et al., 2018; Scharping et al., 2021).

Pyroptosis, a type of proinflammatory cell death, is executed by the gasdermin family of pore-forming proteins (Zhou et al., 2020a). Pyroptosis can lead to the continuous expansion of cells until the cell membrane ruptures and is associated with the release of cell contents and inflammatory response (Shi et al., 2015; Xia et al., 2019). Increasing evidence indicates a close relationship between pyroptosis and tumors. The occurrence of pyroptosis is often accompanied by multiple signaling pathways and inflammatory responses that may be associated with the regulation of tumor immune and is involved in tumorigenesis, antitumor immune function, and chemosensitivity (Man, 2018; Xia et al., 2019; Hou et al., 2020; Tan et al., 2020; Wu et al., 2021c). Interestingly, several studies suggest that hypoxia causes pyroptosis through multiple potential pathways, which may due to the production of reactive oxygen species (ROS) or the mediation of circ-Calm4/mir-124-3p/Pdcd6 axis (Yu et al., 2019; Jiang et al., 2021). Hypoxia-inducible factor 1-α (HIF-1A) is a major transcriptional regulator of cell and developmental responses to hypoxia (Iyer et al., 1998). The activity of HIF-1A is directly regulated by cellular oxygen levels (Semenza, 1999). Jorgensen et al. (2016) found mitochondria with collapsed cristae in pyroptotic cells. Mitochondria are the main sites of ROS production. They have a four-layer structure, and in the process of oxidative phosphorylation (OXPHOS), ROS production mainly occurs on the electron transport chain (ETC) inside the mitochondrial membrane, which folds inward to form ridges, known as cristae (Li et al., 2013). Therefore, collapsed cristae may lead to reduced production of reactive oxygen species (Madamanchi and Runge, 2007), leading to hypoxia of cells. In fact, hypoxia can lead to pyroptosis (Jiao et al., 2018). TNF induced apoptosis has been observed in cell lines of multiple cancer types under normoxic conditions, while the same treatment induces necrotic cell death under hypoxia. It was found that necrotic cell death depended on Caspase-8 and GSDMC, suggesting that increased GSDMC expression under hypoxia could transform caspase-8-induced apoptosis into pyroptosis (Tsuchiya, 2021). A recent study showed that nuclear PD-L1 was found to enhance the expression of GSDMC under hypoxia conditions, while breast cancer patients with increased GSDMC expression had a poor prognosis, suggesting that GSDMC in the center of hypoxia regions may promote tumor progression by inducing the chronic tumor necrosis (Hou et al., 2020). Although pyroptosis has attracted considerable attention because of its potential effect on the tumor immune response, the relationship between hypoxia and pyroptosis and the role of pyroptosis in OS is unclear.

In his study, we established and validated a hypoxia risk model and a pyroptosis scoring model to predict prognosis, changes in the tumor immune microenvironment, and treatment sensitivity. Additionally, we categorized patients with OS into two cluster types based on pyroptosis-related genes that could predict prognosis and immune infiltration. These studies may help assist clinicians in their decision-making and provide insight into the relationships among hypoxia, tumor immune microenvironment, pyroptosis, and OS progression.

## Materials and methods

### Acquisition of datasets

The RNA sequence and osteosarcoma patient characteristics were collected from the TARGET database. RNA expression data of normal human tissues were obtained from the GTEx. The “sva” R package (version 3.8) was used to correct biases after TARGET and GTEx data sets had been pooled. The merged data were used as the training dataset. The external validation GSE cohorts were selected from GEO, named GSE21257 (hypoxia), with detailed survival data. In the establishment of the pyroptosis risk model, TARGET data was separated into two parts: 70% used were for the training dataset, and 30% for the testing dataset. Perl scripts were used to transform the names of microarray probes into gene names.

### Identification of differential genes and functional analysis

Differentially expressed genes (DEGs) related to hypoxia and pyroptosis in normal tissues and osteosarcoma were identified via the “limma” package (version 3.48.3) with logFC >1 and *p* < 0.05 as the criteria. The protein-protein interaction (PPI) network was constructed with the online-supported tool STRING (version 11.5) (Szklarczyk et al., 2015). The ClusterProfiler” package in R software was used to execute the gene ontology (GO) and Kyoto Encyclopedia of Genes and Genomes (KEGG) pathway analyses. The heatmap was generated using the “Pheatmap” package (version 1.0.12).

### Construction and validation of the hypoxia-risk model

To evaluate the expression of hypoxia, hypoxia-related genes (HRG) were obtained from hallmark-hypoxia gene sets obtained from the gene set enrichment analyses (GSEA). The statistically significant DEGs related to hypoxia were tested using univariable and multivariable Cox regression. The hypoxia risk score formula is as follows:

$$Risk\;Score=\overset{N}{\underset{i=1}{\sum }}\left(Coefficient_{i}\times Expression_{i}\right)$$ (1)

### The establishment of the pyroptosis-related signature and survival analysis

Preliminary screening of the survival-related genes was performed by univariate Cox regression analysis, and an optimum pyroptosis-related signature with robust distinguishing ability was established according to the optimum *λ* value via LASSO Cox regression analysis. The following formula was used to calculate the hypoxia risk score for each patient:

$$Riskscore=\overset{N}{\underset{i=1}{\sum }}\left(Coefficient_{i}\times Expression_{i}\right)$$ (2)

### Gene set enrichment analyses and survival analysis

GSEA was utilized to examine the significant difference between the high hypoxia risk and low hypoxia risk groups of the expressed set of genes via GSEA software (version 4.1.0). Enrichment of MSigDB Collection (c7. all.v7.4. symbols) provided by the Molecular Signatures Database was regarded as the reference gene set. Kaplan-Meier survival curves were used for the survival analysis through the “survival” and “survminer” packages in R. Identification of potential prognostic factors was performed by Univariate Cox analysis. The multivariate Cox analysis was used to further determine independent prognostic factors. Time-dependent receiver operating characteristic (ROC) curves were used to determine the predictive efficiency of the model via the survivalROC R package.

### Single sample GSEA and CIBERSORT analysis

To quantify the relative infiltration of various immune cell types and immune-related pathways in the high hypoxia risk and low hypoxia risk groups, ssGSEA analysis was performed.

The enrichment score determined by ssGSEA represents the level of immune-related cell infiltration in each sample. The CIBERSORT tool distinguished 22 types of immune cells via a gene signature matrix including 547 genes, named LM22 ((Newman et al., 2015)). To identify the characteristics of immune status related to pyroptosis, the infiltration of various immune cells in different groups was evaluated by CIBERSORT (version 1.0649).

### Analysis of drug sensitivity

The NCI-60 database was obtained from the CellMiner web site (https://discover.nci.nih.gov/cellminer), which covered 9 cancer categories and 60 different cancer cell lines. Besides, Pearson correlation analysis was used for exploring the relevance between the independent prognostic PRGs and drug sensitivity. Drugs used in this sensitivity analysis are those approved by the FDA or those in clinical tests.

### RNA interference

Two pairs of Small interfering RNA (siRNA) targeting human *CCL28/HMGB1/KLF2/TLR4/TNFSF18* were synthesized by Invitrogen, named Gene-SiRNA1and Gene-SiRNA2. The primer sequences are provided in Supplementary Table S1. Osteosarcoma cells were seeded with a density of (Rosen, 1985; Ritter and Bielack, 2010; Li et al., 2011; Goguet-Surmenian et al., 2013; Smeland et al., 2019) × 10^5^ per well in 12-well plates. According to the manufacturer’s instruction, transfection of siRNAs was carried out using Lipofectamine™ 3,000 transfection with the when cells reached 80% confluence. Total RNA was extracted with TRIzol reagent and reverse transcribed to cDNA. Based on mRNA levels determined by RT-qPCR analysis. The most effective siRNA was determined. This experiment was carried out with 2 replicates.

### Detections of cell apoptosis by flow cytometry

Transfection was conducted as describe above and the supernatant was collected after 48 h of incubation. Subsequently, cells were digested by trypsin without EDTA and harvested the single cell suspension. The cells were resuspended with cold PBS and cell precipitates were collected by centrifugation. After washing with binding buffer, cells were stained with Annexin V−FITC and propidium iodide. Then, apoptotic analysis was immediately performed by flow cytometer.

### Immunofluorescence

First, paraffin tumor sections were dewaxed in water. Then, a microwave thermal process was used to repair the antigen. The slides were washed thrice in phosphate buffer solution (PBS) for 5 min each time, blocked with 10% goat serum, and incubated in primary antibody at 4°C overnight, then added secondary antibodies for incubated 50 min at room temperature. The nuclei were stained by DAPI solution after three times wash and incubated 10 min. Sections were then observed and images were collected using a fluorescence microscope (NIKON ECLIPSE C1). The paraffin tumor sections were obtained from Wuhan Sevier Biotechnology Co., Ltd.

### Data analysis

R software (version x64 4.1.0) was used for the statistical analysis. Correlations between variables were analyzed using Pearson or Spearman coefficients. Comparisons between the two groups were performed using the Wilcoxon test. The Kruskal Wallis test was conducted to compare more than two groups. The Kaplan-Meier analysis was carried out *via* “survival” and “survminer” packages in R to compare the overall survival between groups. All statistical *p* values were two-sided, and the level of significance was set at *p* < 0.05.

## Results

### Hypoxia risk model to predict osteosarcoma prognosis

**FIGURE 1:**
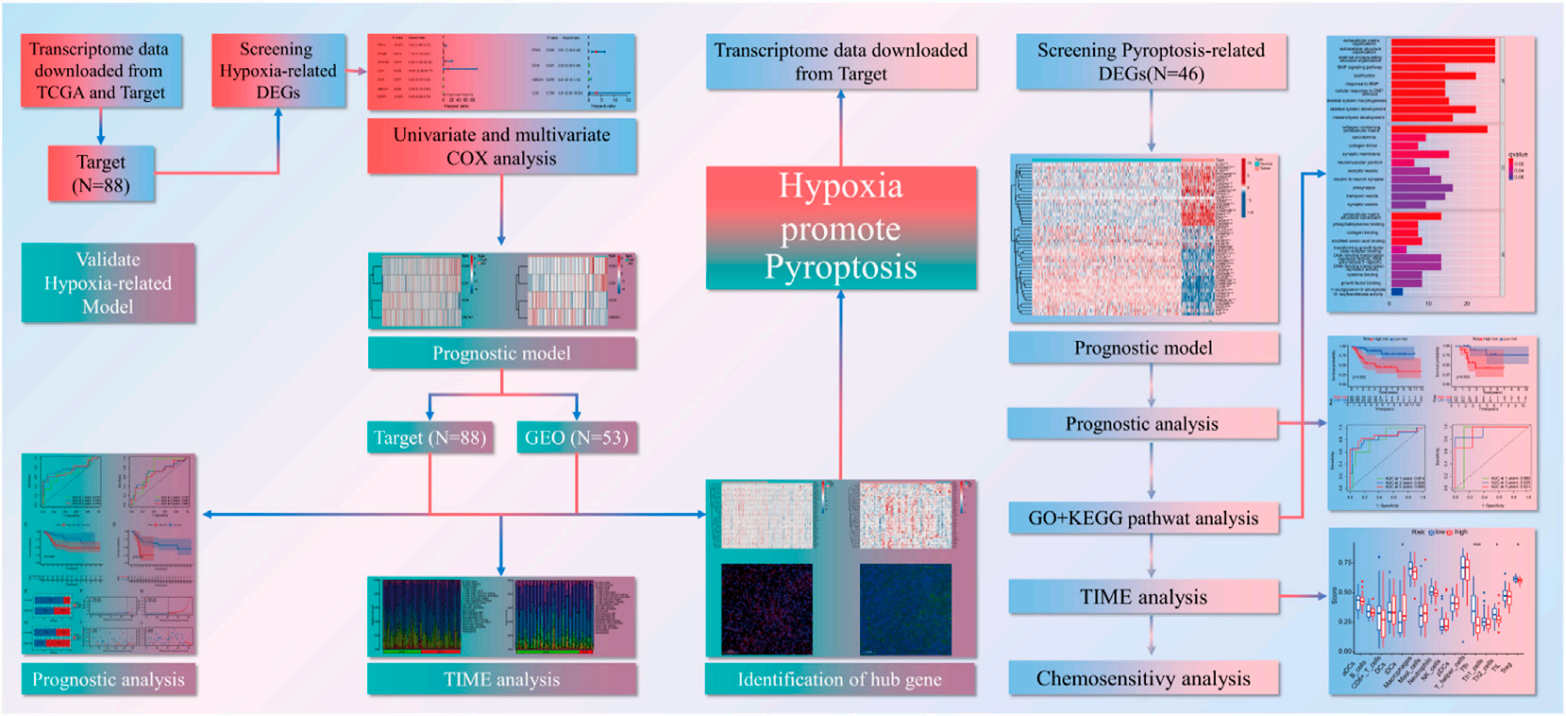
Flow chart of experimental design.

**FIGURE 2:**
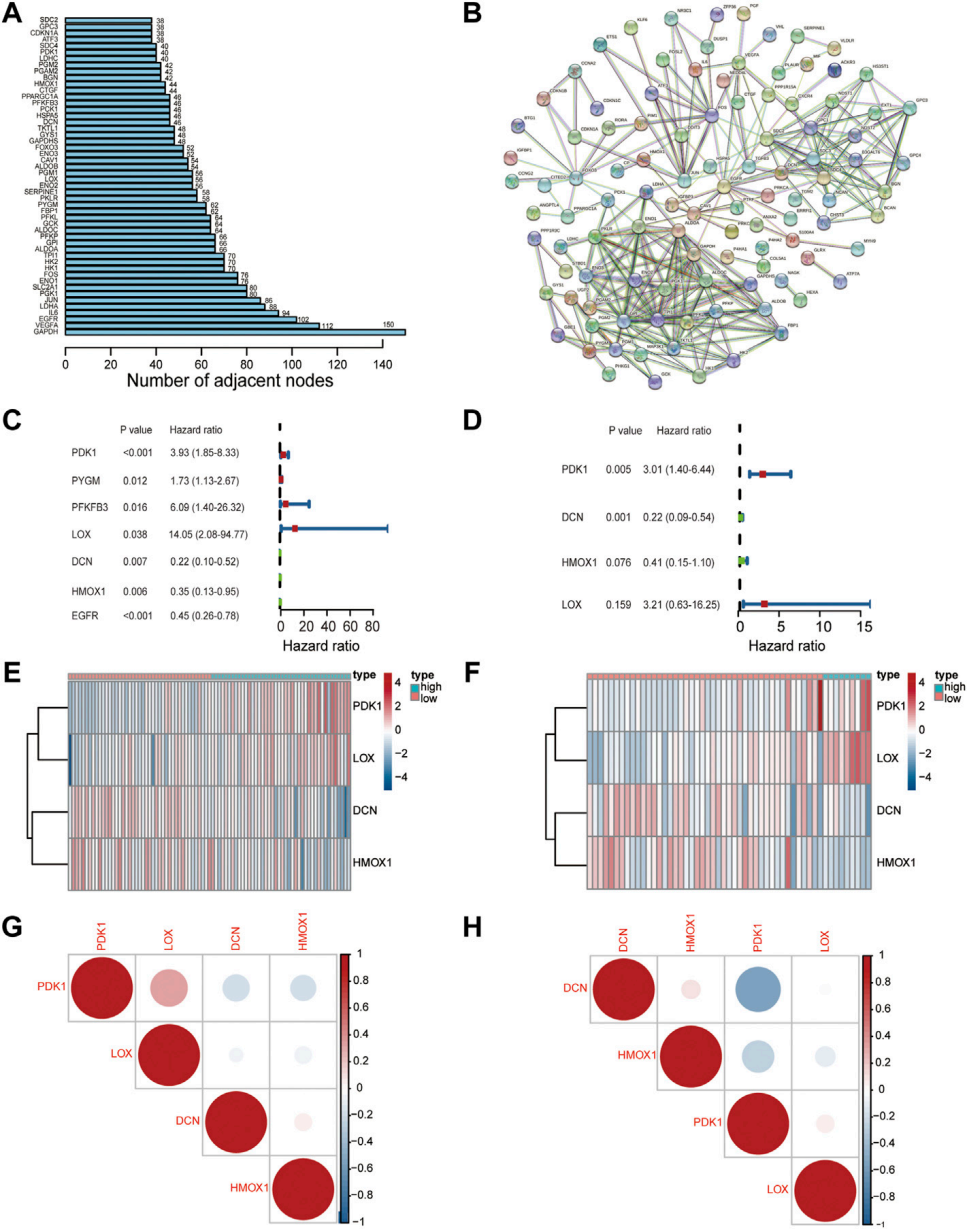
Characterization of a hypoxia risk model to predict prognosis of OS. **(A)** The number of adjacent nodes per hypoxia-related gene; **(B)** PPI network indicating the correlation of HRGs; **(C,D)** A hypoxia-risk model was evaluated by univariate and multivariate Cox regression; **(E,F)** Heatmap displaying expressions of hypoxia-related DEGs and comparison between high and low hypoxia risk groups in TARGET **(E)** and GEO databases **(F)**; **(G,H)** Correlation analysis of hub gene in TARGET **(G)** and GEO databases **(H)**.

The flow chart illustrates the entire study design (Figure 1). Two hundred hypoxia-related genes (HRGs) were downloaded from GSEA software. We evaluated the number of adjacent nodes and identified the top 50 genes with most interactions that might play a significant role in the response to hypoxia (Figure 2A). A more detailed PPI analysis was conducted to visualize the internal links among these HRGs (Figure 2B).

Univariate Cox regression analysis was first performed to screen out prognostic genes in the training set that was merged with the TARGET and GTEx datasets (Figure 2C). Then, four genes (*PDK1, LOX, DCN,* and *HMOX1*) were selected to construct an optimal predictive model by utilizing the multivariate Cox regression analysis (Figure 2D). The formula for the hypoxia risk score calculation is as follows:

Riskscore=1.10×PDK1+1.17×LOX+(−1.53)DCN+(−0.90)HMOX1 (3)

A heatmap of the four genes expression was used to visualize the different expression patterns. *PDK1* and *LOX* were upregulated in the high hypoxia risk score group in both TARGET (training) (Figure 2E) and GEO (testing) datasets (Figure 2F). Correlations among all four genes were evaluated and were found not to be significant in both the dataset and the testing GEO dataset (Figures 2G,H). To determine the prognostic value of the four genes, overall survival curves for patients with different levels of individual gene expression in the training dataset were generated. The results indicated that higher *PDK1* expression was associated with low survival probability (*p* = 0.002, Supplementary Figure S1), while there was no difference between the high- and low-expression groups for the other genes. The above results suggested that these four HRGs could be used to build the hypoxia risk model.

### Analysis of GO enrichment and KEGG pathway enrichment

**FIGURE 3:**
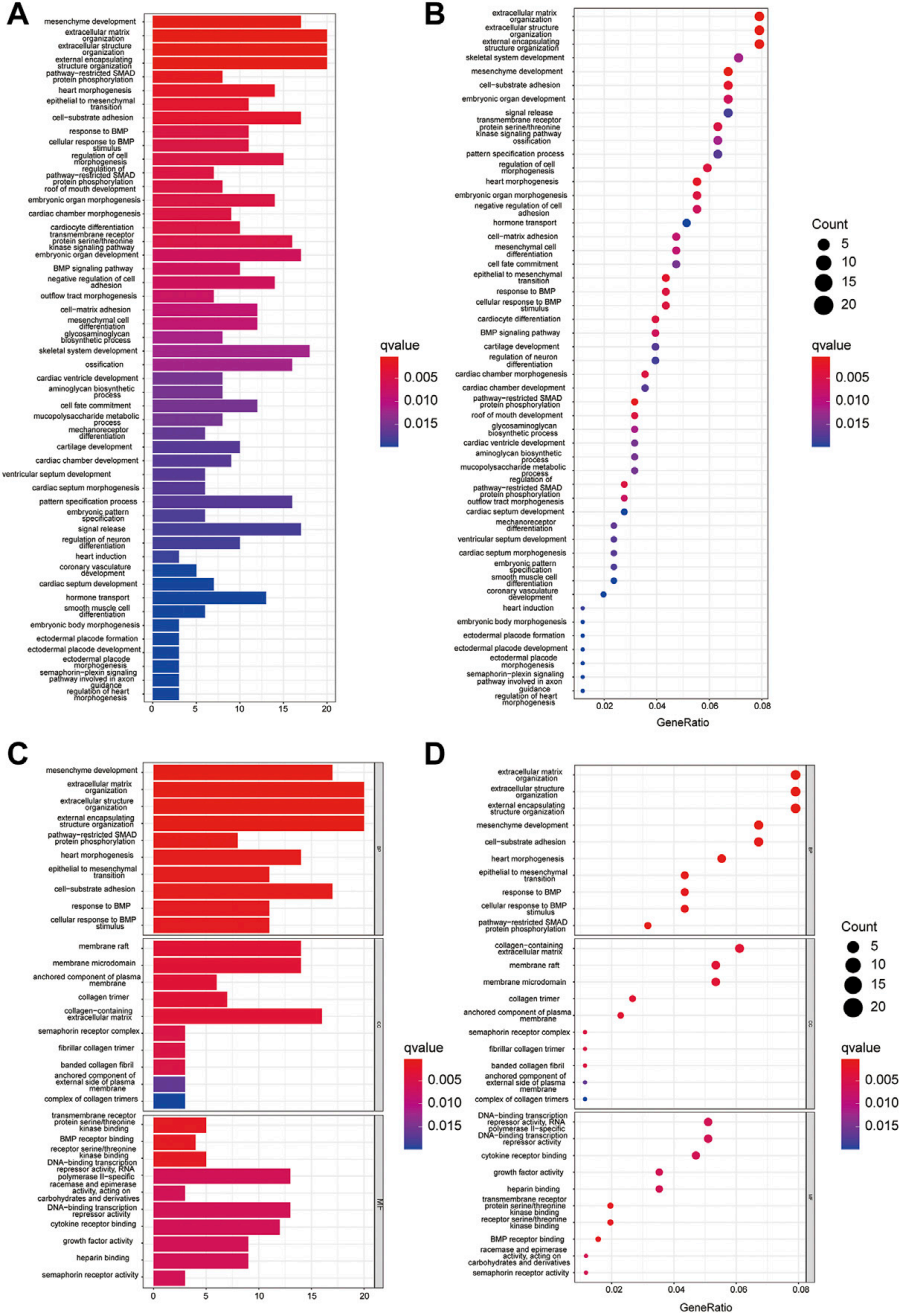
GO and KEGG enrichment analysis in TARGET dataset. **(A,C)** The KEGG **(A)** and GO **(C)** analysis by bar graph; **(B,D)** Bubble chart shows significantly enriched KEGG **(B)** pathway and GO **(D)**.

To further explore the signaling pathways for the HRGs, KEGG pathway enrichment (Figures 3A,B) and GO enrichment (Figures 3C,D) were conducted using TARGET dataset. The results indicated that the HRGs were primarily associated with mesenchyme development, extracellular matrix or structure organization, external encapsulating structure organization.

### Prognostic value of the hypoxia risk model in OS

Hypoxia often correlates with cancer progression and poor survival outcomes; thus, we further examined the predictive ability of the hypoxia risk model. Based on the median value of the hypoxia risk score, patients were assigned to the high or low hypoxia risk group in the TARGET and GEO datasets.

**FIGURE 4:**
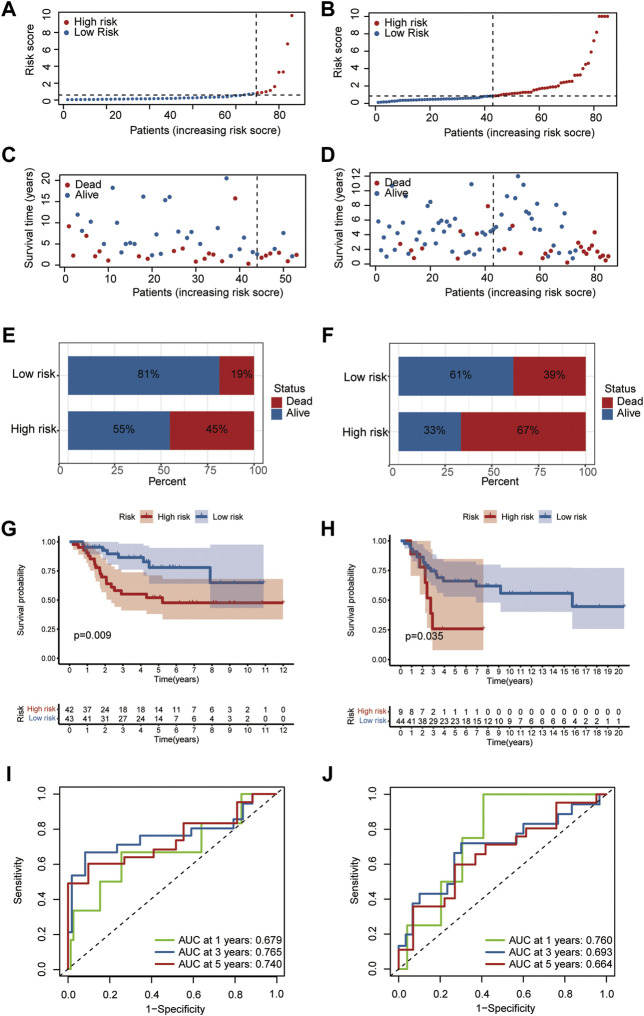
Predictive ability of the hypoxia risk model in OS. **(A,C)** Distribution of survival status and risk score in the high- and low-risk groups in TARGET dataset; **(B,D)** Distribution of survival status and risk score in the high- and low-risk group in the GEO database; **(E,F)** Mortality rates of the high- and low-risk group in TARGET **(E)** and GEO **(F)** databases; **(G,H)** The curves for predicting overall survival for patients in TARGET **(G)** and GEO **(H)** databases; **(I,J)** ROC curves of TARGET **(I)** and GEO **(J)** cohorts.

Patients in the training (Figure 4A) and testing datasets (Figure 4B) were stratified into high and low groups according to the median value of the HRS. The results showed that the mortality of the high hypoxia risk group was significantly higher than that of the low hypoxia risk group in training dataset (Figures 4C,E) and testing dataset (Figures 4D,F). Then, Kaplan-Meier analysis was performed to further assess the predictive ability of the hypoxia risk model in patients with OS. The results showed that the overall survival was worse in the high hypoxia risk group than in the low hypoxia risk group in the TARGET dataset (Figure 4G, *p* = 0.009); this was further confirmed in the GEO cohort (*p* = 0.035, Figure 4H). Then, the ROC curves were obtained for the TARGET and GEO cohorts to assess the predictive accuracy of the hypoxia risk model for the 1-, 3- and 5- year survival rates. In the TARGET dataset, the area under the ROC curve (AUC) was 0.679 at 1-year, 0.765 at 3-year, 0.740 at 5-year, indicating the robustness and accuracy of the hypoxia risk model (Figure 3I). This was confirmed in the GEO dataset (Figure 4J). Therefore, the hypoxia risk model is closely related to OS prognosis.

### Immune infiltration of high- and low-hypoxia risk OS patients

**FIGURE 5:**
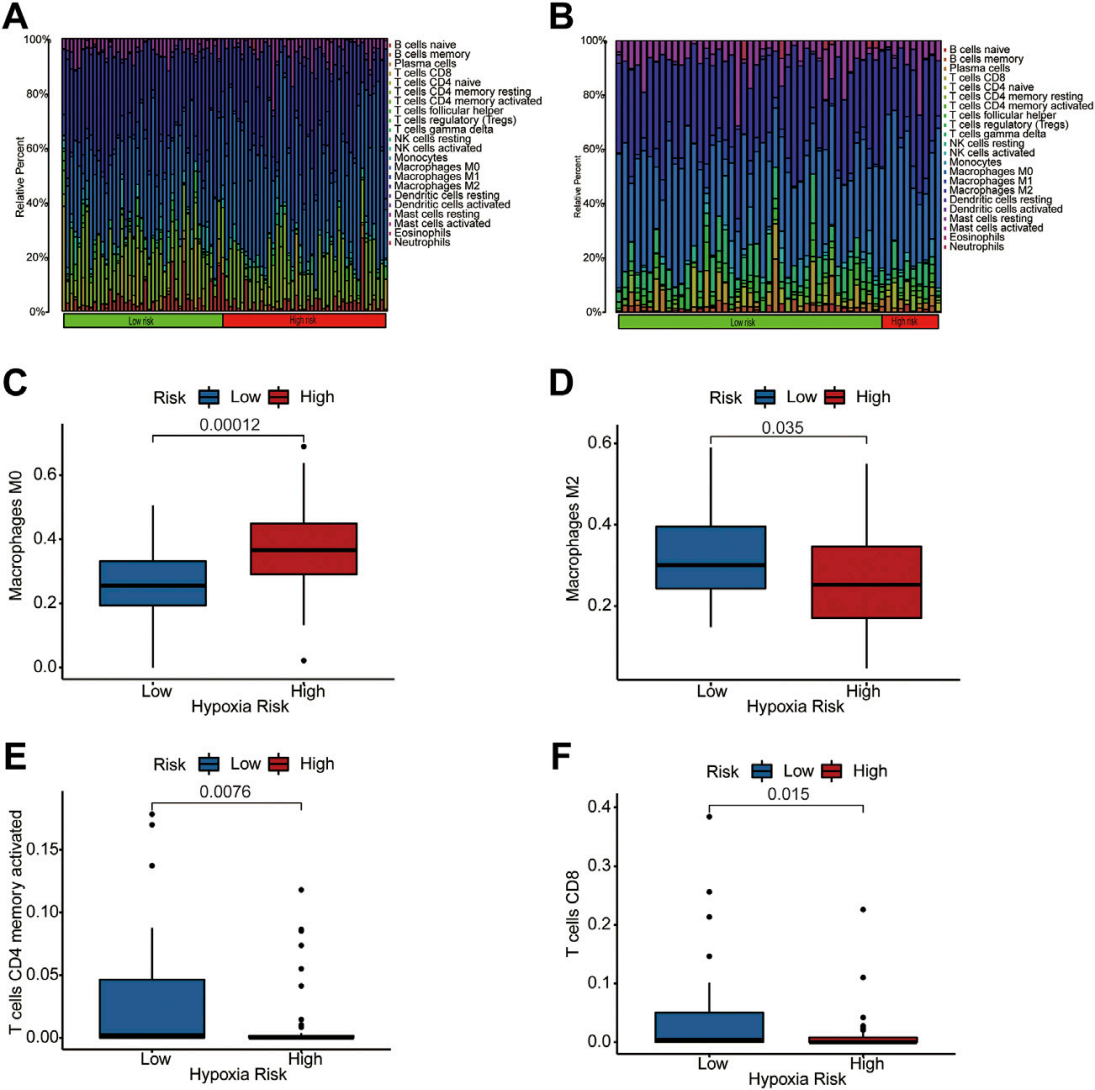
The immune landscape of two hypoxia score subtypes. **(A,B)** Distribution of immune infiltration in patients of high- and low-hypoxia risk groups from TARGET **(A)** and GEO **(B)** databases; **(C–F)** Comparison of various immune cells between high- and low-hypoxia risk groups from training dataset, visualized by Box plots.

Previous studies indicated that the microenvironment in hypoxic conditions may help tumors resist antitumor immunity by reshaping TIM and enhancing immune evasion. We performed a CIBERSORT analysis to identify immune composition differences between the high- and low-risk groups. The heatmap of immune composition in patients from the training and testing datasets is shown in Figures 5A,B. The results suggest a lower immune cell infiltration in high hypoxia risk patients from the TARGET dataset such as CD8^+^ T cells, activated memory CD4^+^ T cells, and macrophage M2 cells, while M0 macrophages showed higher infiltration in the high hypoxia risk score group in the TARGET dataset (Figures 5C–F). Furthermore, the expression of Granzyme B (*GZMB*) and Interferon Gamma (*IFNG*) negatively correlated with hypoxia risk score in TARGET dataset (Supplementary Figures S2A, B) and GEO dataset (Supplementary Figures S2C, D). These results show that hypoxia may participate in TIM rebuilding and could be a target for immune therapy.

### High hypoxia risk correlates with the immunosuppressive microenvironment

**FIGURE 6:**
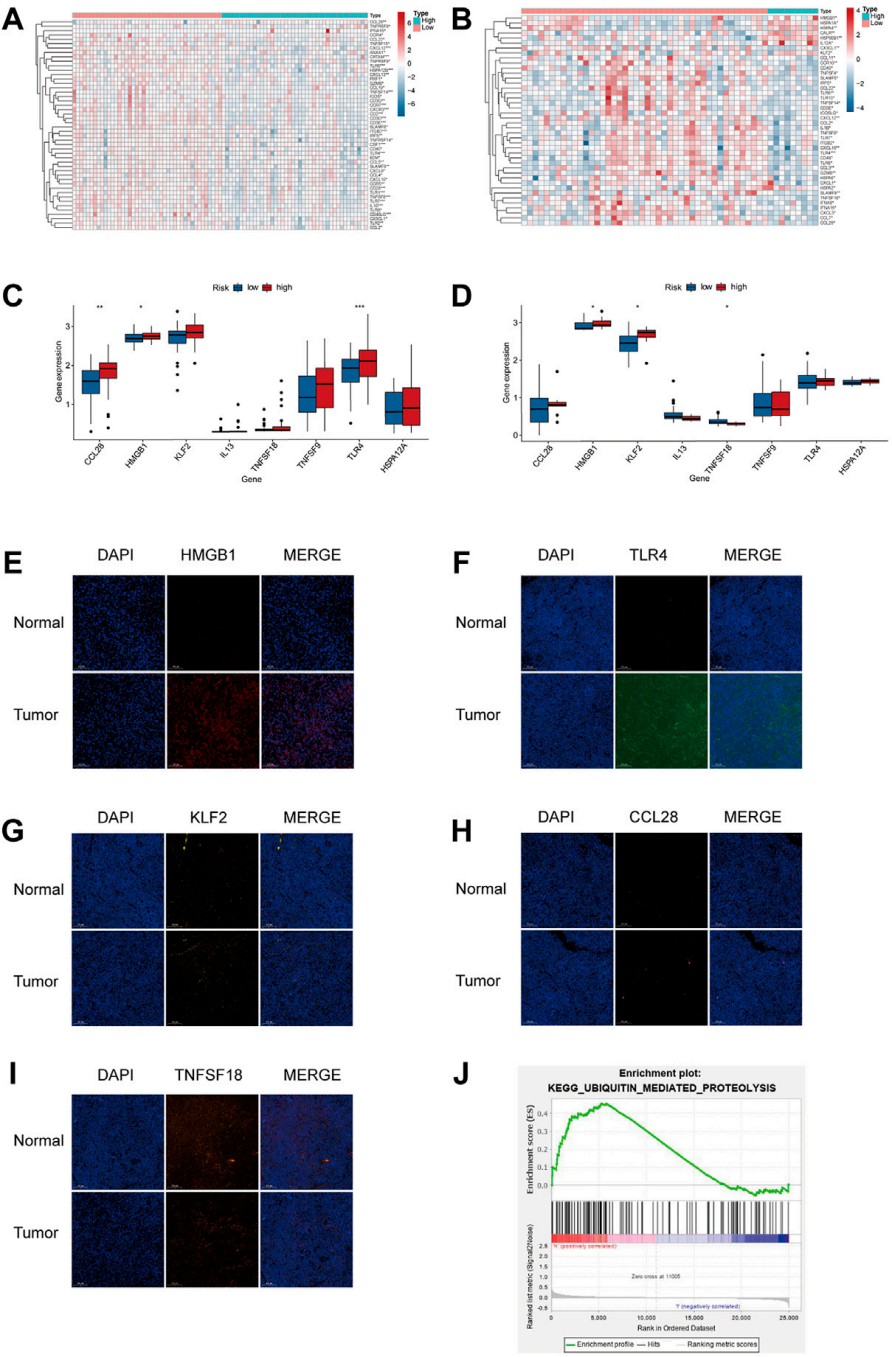
TIM of the high hypoxia risk group exhibited immunosuppressive characteristics. **(A,B)** Heatmap showing the positively regulated genes of the Cancer-Immunity Cycle in two subtypes in the training **(A)** and testing **(B)** databases. **(C,D)** Expression of immune-related signature in the training and testing dataset. **(E–I)** Expression of immune-related regulators were verified by immunofluorescence in tumor and normal tissues. **(J)** GSEA in TARGET cohort. ***p < 0.001.

The cancer-immunity cycle has guided studies of immune therapy and describes a series of stepwise events for an antitumor immune response such as antigen release and activation, immune cell migration, identification, and death of cancer cells (Chen and Mellman, 2013). To investigate the relationship between hypoxia and the genes that positively regulate these processes, we downloaded gene signatures from Tracking Tumor Immunophenotype (http://biocc.hrbmu.edu.cn/TIP/) (Xu et al., 2018). As shown in Figure 6, most positively regulating genes of the cancer-immunity cycle were downregulated in the patients with a high hypoxia risk score, suggesting that hypoxia is associated with low activities of these antitumor immune processes.

Based on the results of the hypoxic microenvironment, we further explored the links between our model and immune-related factors. As shown in Figure 6A, the high hypoxia risk group had high expression of *HMGB1* and *TLR4* in the TARGET dataset. In the GEO dataset, the *HMGB1* and *KLF2* were highly expressed in the high hypoxia risk group, the expression of *TNFSF18* was lower in the high hypoxia risk group than in the low hypoxia risk group (Figure 6B). Immunofluorescence was determined in tumor and normal tissues to further verify the immune regulator expression. As shown in Figures 6E–I, the expression of *HMGB1* and *TLR4* was significantly high in tumor tissue compared with that in normal tissue, the expression of *CCL28*, *KLF2* and *TNFSF18* in tumor were similar to normal tissues. In an attempt to investigate the potential pathway related to hypoxia, we conducted GSEA and found that the ubiquitin-mediated proteolysis pathway was enriched in high hypoxia risk patients in the TARGET dataset (Figure 6J).

The above results demonstrated that hypoxia might participate in establishing a TIM with immunosuppression by affecting immune regulators.

### Knockdown of HMGB1 and TLR4 promoted apoptosis of osteosarcoma cells

**FIGURE 7:**
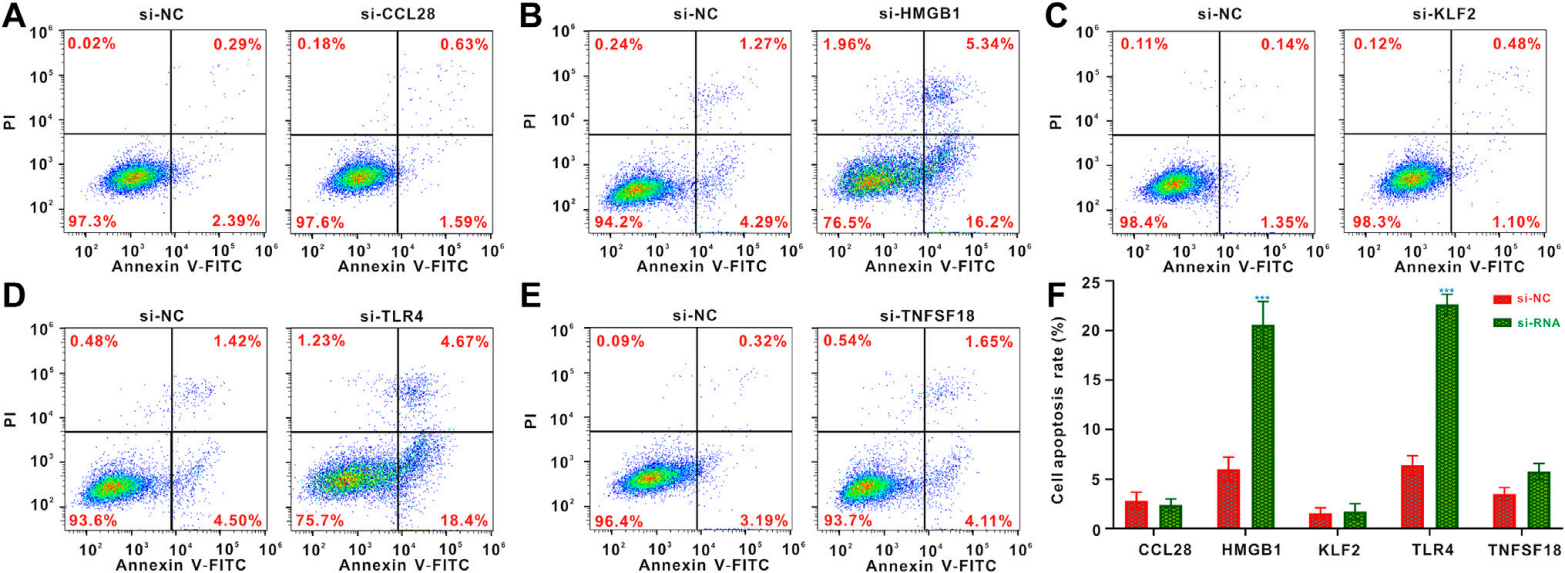
Flow cytometric analysis of cell apoptosis. **(A–E)** Flow cytometric analysis for osteosarcoma cells following *CCL28*, *HMGB1*, *KLF2*, *TLR4* and *TNFSF18* knockout **(F)** Quantified results of the flow cytometric analysis. *p < 0.05; **p < 0.01; ***p < 0.001.

To identify the effects of *HMGB1*, *TLR4*, *CCL28*, *KLF2* and *TNFSF18* knockdown on apoptosis of osteosarcoma cells, the flow cytometry analysis was conducted. The result indicated that *CCL28*, *KLF2* and *TNFSF18* did not significantly affect cell apoptosis (Figures 7A,C,E,F), while the knockdown of *HMGB1* and *TLR4* significantly increased the proportion of apoptotic of osteosarcoma cells (Figures 7B,D,F).

### Identification of DEGs between healthy and tumor tissues

**FIGURE 8:**
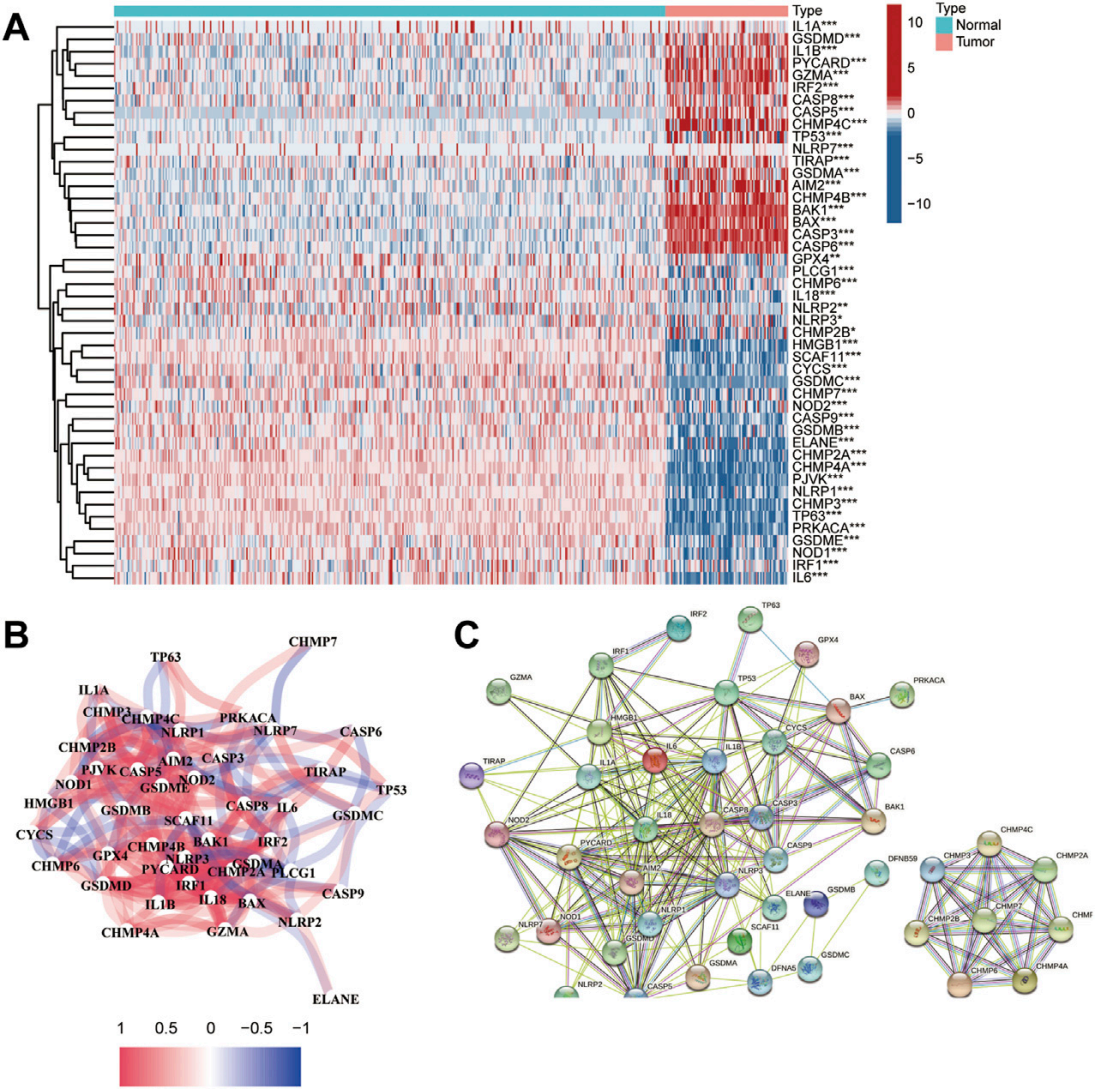
Identification of DEGs between healthy and tumor tissues. **(A)** Heatmap of the PRGs expressed in normal and tumor tissues; **(B)** The network of the PRGs showing interactions among the genes; **(C)** PPI network of the PRGs.

According to previous evidence of the close relationship between immune regulators and pyroptosis, for example, HMGB1 and TLR4 may promote the development of pyroptosis (Wang et al., 2019a; Wang et al., 2020a). To determine whether there was any association between the hypoxic microenvironment and pyroptosis, we identified 46 DEGs by comparing the levels of 52 pyroptosis-related genes (PRGs) in the training dataset. The differential levels of gene expression in tumor and normal tissues were analyzed using a heatmap (Figure 8A). As shown in the heatmap, *IL1A, GSDMD, IL1B, PYCARD, GZMA, IRF2, CASP8, CASP5, CHMP4C, TP53, NLRP7, TIRAP, GSDMA, AIM2, CHMP4B, BAK1, BAX, CASP3,* and *CASP6* showed upregulated expression patterns, while other genes were expressed at low levels in patients with OS. The correlation network of the DEGs is presented in Figure 8B. Moreover, we analyzed the PPI network using the STRING online tool (Figure 8C).

### Classification of OS patients based on the DEGs

**FIGURE 9:**
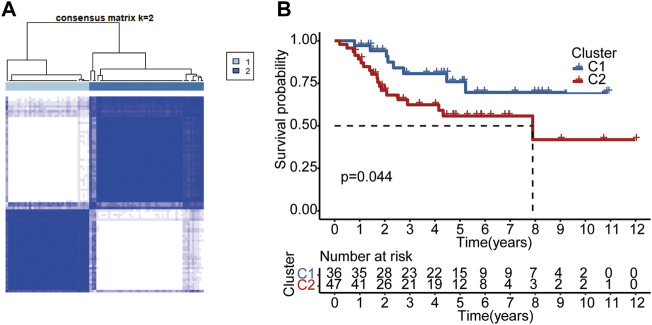
Subtypes of OS according to DEGs. **(A)** Based on the consensus clustering matrix, OS patients were divided into two clusters (k = 2). **(B)** Kaplan-Meier analysis of the two clusters.

A consensus clustering analysis in the TARGET dataset was conducted to further understand the link between PRG and OS. According to the level of pyroptosis-related DEGs expression, we determined that the intergroup correlations were the lowest and the intragroup correlations were the highest, while the clustering variable (k) value was 2, suggesting that patients could be classified into two separate clusters (Figure 9A). We then compared the overall survival times of the two clusters. As shown in Figure 9B, cluster 1 was associated with a higher survival advantage than cluster 2. The analysis results for different k values are shown in Supplementary Figure S3.

### Establishment and validation of pyroptosis signature

**FIGURE 10:**
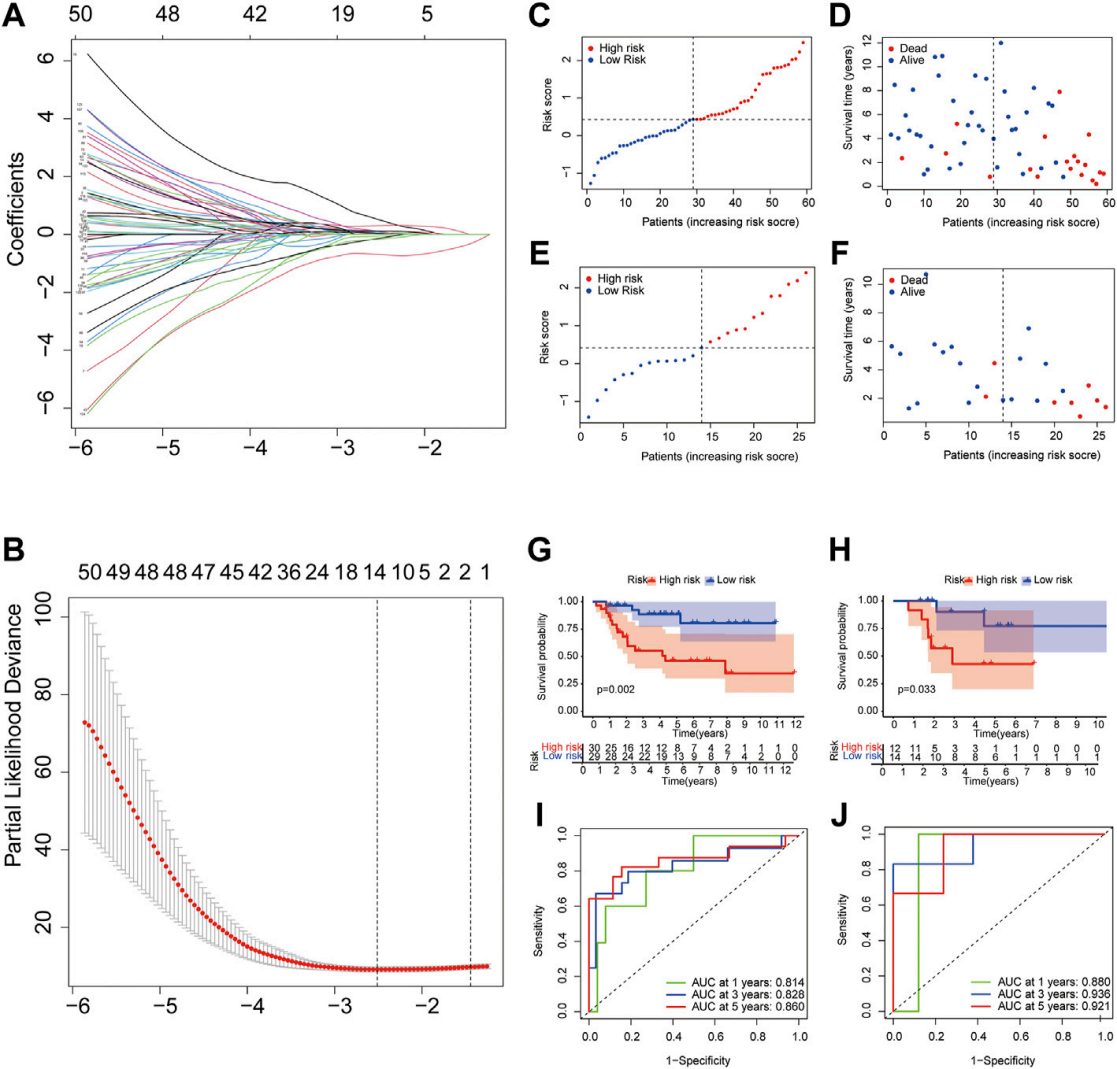
Construction and validation of pyroptosis risk signature. **(A,B)** LASSO regression and cross-validation of genes; **(C,E)** OS patients assigned in subtypes by the median value of the pyroptosis risk score in training **(C)** and testing cohorts **(E)**; **(D,F)** Distribution of survival status in the high and low pyroptosis risk group from training cohort; **(G,H)** Overall survival curves for patients with high and low pyroptosis risk scores in training **(G)** and testing cohorts **(H)**; **(I,J)** ROC curves of the pyroptosis risk signature indicating the predictive ability in training **(I)** and testing cohorts **(J)**.

For preliminary screening of the PRGs, univariate Cox regression analysis was used in the TARGET dataset (Supplementary Figure S4). A group of 14 genes (*HSD11B2, KIF25, BST1, SNORA75, GBP1, CLDN11, ZNF692, ARMC4, FPR1, PCDHB6, IHH, TPD52, RAMP1,* and *TAC4*) was classified using the optimal *λ* value (Lossos et al., 2004) by performing the LASSO Cox regression analysis (Figures 10A,B). The coefficient values for each gene are presented in Supplementary Table S2.

To evaluate further the prognostic value of the pyroptosis risk model, we assigned patients into low and high pyroptosis risk score groups in the training and testing cohorts, applying the cut-off median value from training (Figure 10C) and testing (Figure 10E) datasets. The results of the principal component analysis showed that patients with different pyroptosis risk scores were divided into two groups in both the training and testing datasets (Supplementary Figure S5).

To compare survival outcomes between the two pyroptosis risk groups, we performed overall survival and survival status analyses in the training cohort. The low pyroptosis risk score group was associated with better survival status (Figure 10D) and higher overall survival time (Figure 10G) than the high pyroptosis risk group; this was validated further by the testing dataset (Figures 10F,H). The ROC curve shows that AUCs were 0.814 at 1-year, 0.828 at 3-year, 0.860 at 5-year points, suggesting that the risk model has a high predictive value in the training dataset (Figure 10I). This was further confirmed by analyzing the testing cohort (Figure 10J).

### Functional analyses of the pyroptosis risk model

To determine the functional enrichment and pathways in groups classified by the pyroptosis risk score, we used R software to identify 172 DEGs conforming to the criteria of FDR <0.05 and |log_2_FC | ≥ 1.

**FIGURE 11:**
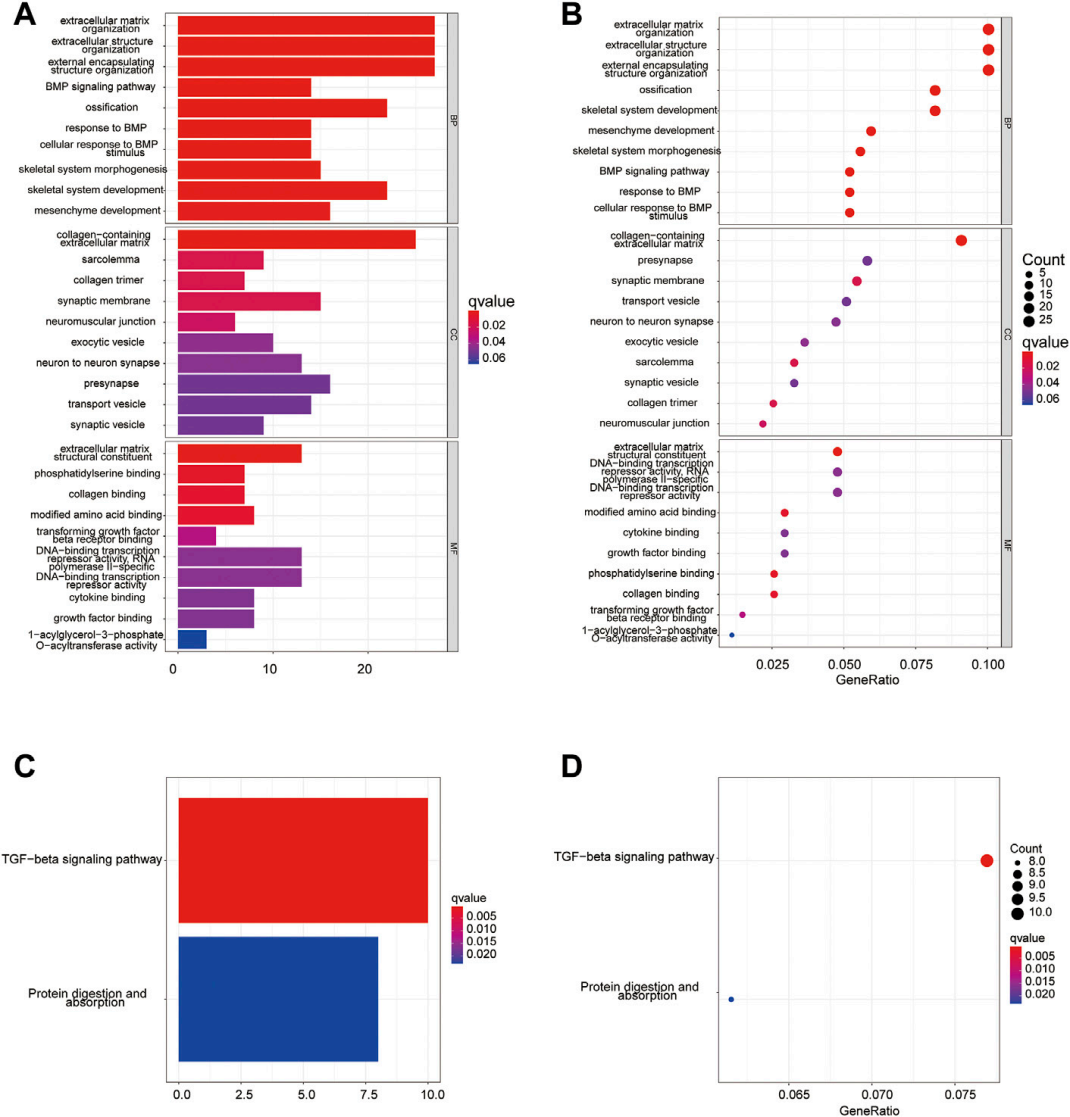
Functional analysis of training cohort (from TARGET dataset). **(A,C)** Barplot graph for GO **(A)** and KEGG **(C)** pathways; **(B,D)** Bubble graph for GO **(B)** and KEGG **(D)** enrichment.

Using these DEGs, we performed the GO (Figures 11A,B) and KEGG (Figures 11C,D) pathway analysis on the training dataset and determined that the DEGs were mainly associated with the extracellular matrix or structure organization, ossification, BMP, TGF-beta signaling pathway and protein digestion and absorption.

### Correlations of the immune activity and pyroptosis risk signature

**FIGURE 12:**
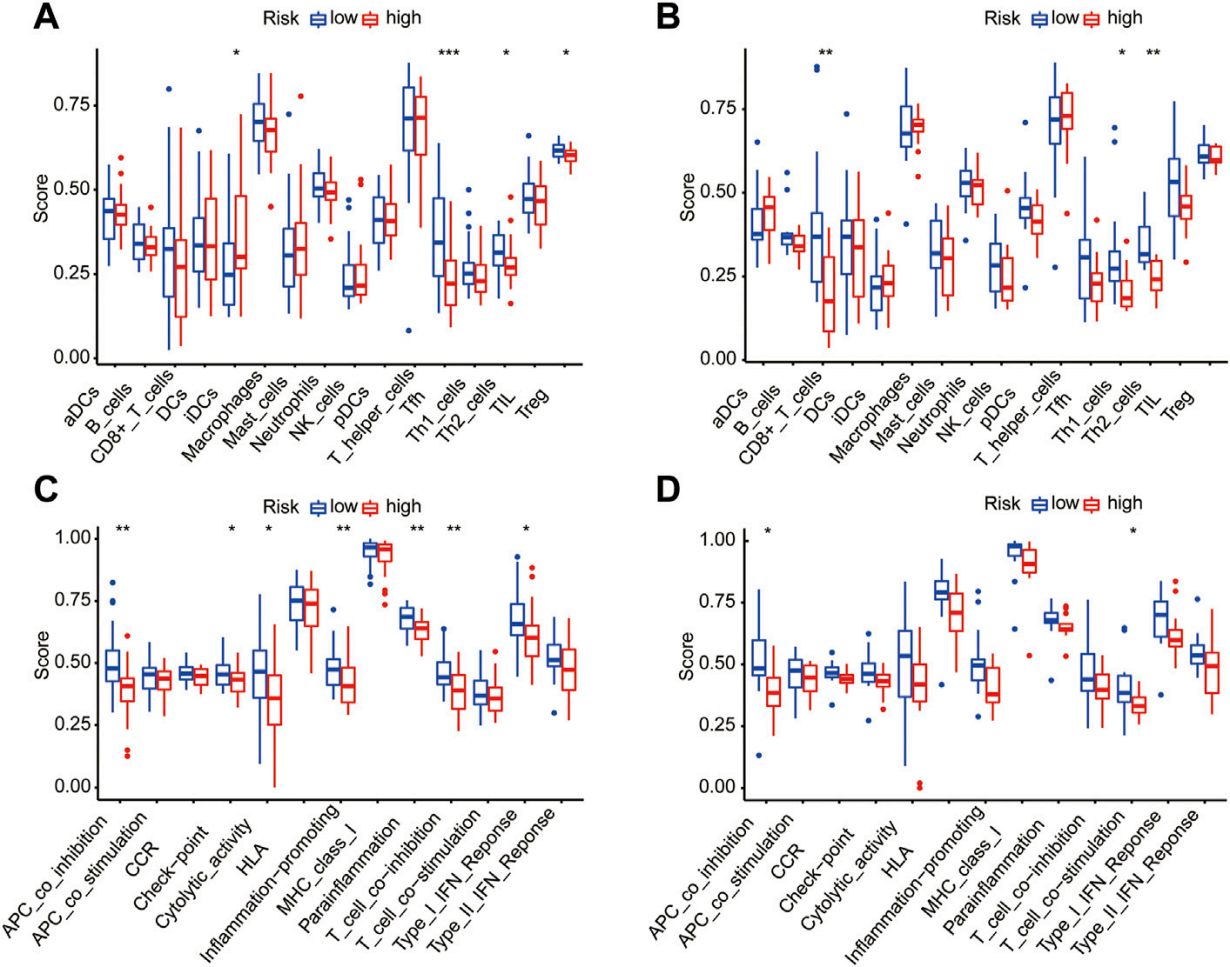
Correlation between immune activities and risk score in training and testing datasets. **(A/B)** Difference of infiltrating immune cell subpopulations and levels between the two pyroptosis risk groups in the training dataset **(A)** and testing cohorts **(B)**. **(C/D)** The relationship between pyroptosis risk score and 13 immune-related pathway activities in the training dataset **(C)** and testing cohorts **(D)**.

To further investigate the potential correlation between the different pyroptosis-related risk and immune activities, we evaluated the level of 13 immune-related pathway activities and the enrichment of various immune cells between the two risk-score groups in the training dataset by ssGSEA (Figure 12A). The results suggested a lower infiltration of immune cells (e.g., IDCs cells, Th1 cells, Th2 cells) in the high pyroptosis risk score group. Moreover, we find lower infiltration of CD8^+^ T cells, Th1 cells and Th2 cells in the high pyroptosis risk score group in the testing dataset (Figure 12B). The high pyroptosis risk score group showed lower activities in some immune pathways such as antigen-presenting cell (APC) co-inhibition and T cell co-stimulation (Figure 12C). These results indicate that a higher pyroptosis score may be linked to a low antitumor immune response; this was also validated by performing the above examination with the testing dataset (Figure 12D).

### Pyroptosis risk signature predicts chemotherapy sensitivity

**FIGURE 13:**
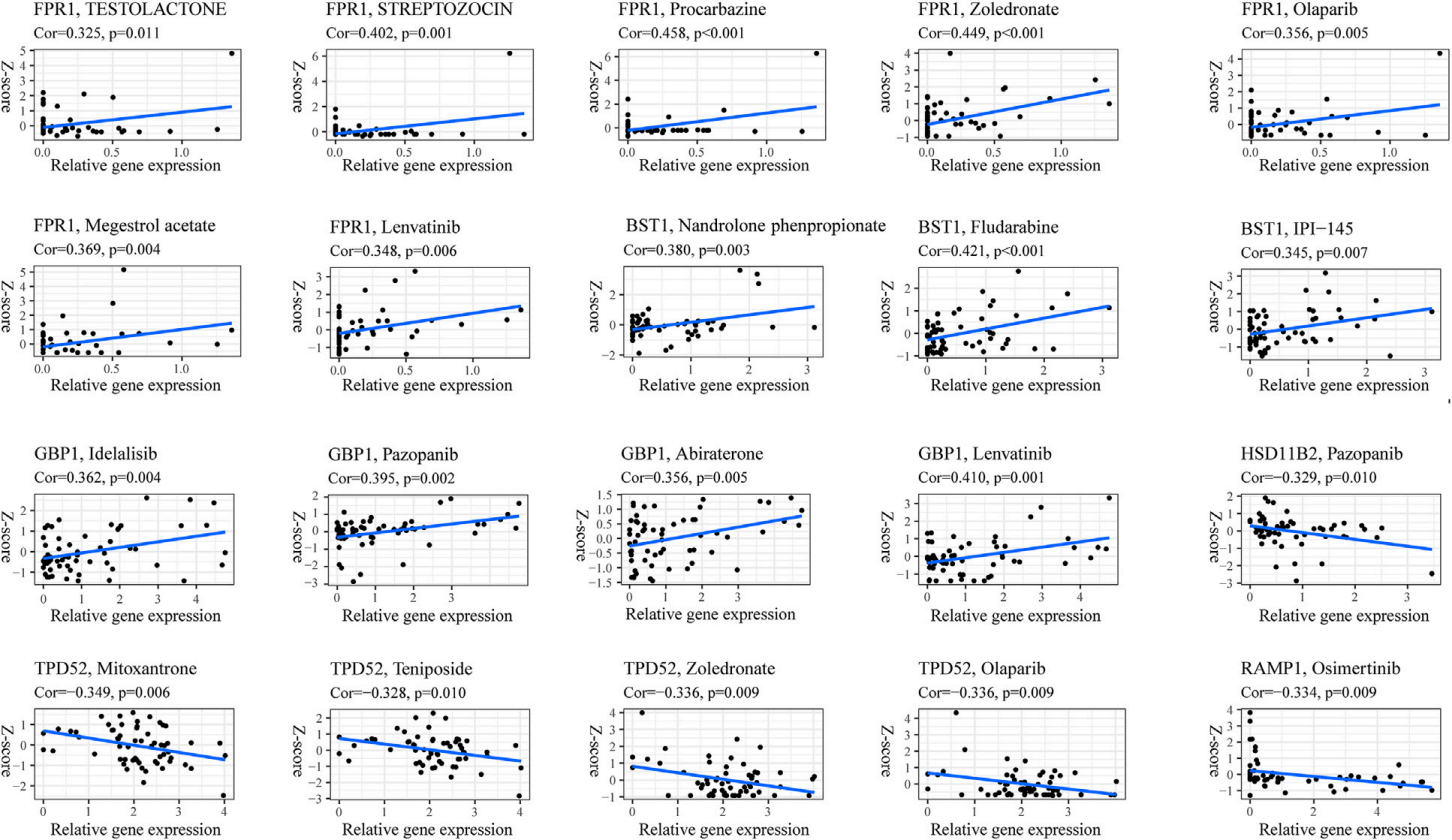
Correlation between pyroptosis score and chemotherapy sensitivities.

Nowadays, with the increase in survival rate due to various chemotherapy combinations, multimodal treatment is considered the most effective strategy. Therefore, we explored whether the expression of the 14 genes pyroptosis risk signature was related to the sensitivity of common chemotherapies in the training dataset. As shown in Figure 13, the expression of *FPR1*, *BTS1* and *GBP1* exhibited a positive correlation with chemotherapy strategies, and the expression of *HSD11B2*, *TPD52* and *RAMP1* exhibited a negative correlation with chemotherapy strategies. The results indicated that pyroptosis might be a potential target for chemotherapy in patients with OS.

## Discussion

Osteosarcoma is a highly aggressive and malignant bone tumor with a poor prognosis that occurs primarily in children and adolescents (Jo and Fletcher, 2014). Over the past half-century, new profound insight and effective strategies have been urgently sought to resolve the clinical limitations because the treatment efficacy of OS has plateaued (Ballatori and Hinds, 2016). Despite the growth of multimodal treatment regimens and diagnosis of OS in previous decades, the survival rate remains low; this could be attributed mainly to the limitations of diagnosis, early metastasis, and low chemosensitivity (Niu et al., 2019). Previous evidence indicates that the alteration of TIM and the development of pyroptosis might play a major role in tumor pathogenesis. However, most studies have focused only on individual correlations between hypoxia, TIM, pyroptosis, and OS, and the characteristics of the internal connections among all these factors have not been systematically evaluated; understanding of these interactions might shed new light on treatment methods.

Hypoxia frequently exists in TIM and has been considered a critical mediator of tumor aggressiveness, metastasis, drug sensitivity, and poor prognosis. In addition, hypoxia has been reported to be directly linked to effects on immunosuppressive cells and molecules (Kumar and Gabrilovich, 2014). Several studies also suggested a close linkage between hypoxia and pyroptosis. Meanwhile, as the awareness of pyroptosis increases, the role of pyroptosis in TIM, antitumor response, and tumor progression has received increasing attention. Based on this experience, we established a hypoxia-risk model linked with a pyroptosis-related risk model to predict prognosis, TIM, and chemoresistance, and to reveal the relationship among hypoxia, TIM, and OS.

We initially created a hypoxia-risk model consisting of four HRGs and established a pyroptosis-risk model with 14 PRGs. It has been demonstrated that pyruvate dehydrogenase kinase 1 (*PDK1*) and lysyl oxidase (*LOX*) were associated with chemoresistance, tumor metastasis, and poor survival (Umezaki et al., 2019; Siu et al., 2020). Our results show high hypoxia risk with high levels of expression of *PDK1* and *LOX*, validating the suggestion that high hypoxia is linked with high *PDK1* and *LOX*, as previously reported (Majmundar et al., 2010; Ji et al., 2013). In contrast, decorin (DCN) plays a role in impeding tumorigenesis (Sainio and Järveläinen, 2019). Heme oxygenase-1(*HOMX1*) is considered to be the main protein in diseases that may be driven by oxidative and inflammatory attacks (Waza et al., 2018). After several survival analyses, this hypoxia risk model showed robust accuracy in predicting tumor prognosis.

In addition to contributing to OS aggression and malignancy, hypoxia promotes tumor escape and immune resistance by reducing the infiltration of immune cells and establishing an immunosuppressive microenvironment that may protect tumors from antitumor immune attacks. Previous studies indicate that T cells, and especially CD8^+^ T cells perform an essential role in the antitumor immune response, for example, CD8^+^ T cells induce ferroptosis and pyroptosis resulting in the inhabitation of tumor growth and enhancement of antitumor immunity (Lang et al., 2019; Tang et al., 2020; Zhang et al., 2020); enrichment of CD4^+^ T cells is associated with good prognosis (Liu et al., 2021). However, CD8^+^ T cells can become rapidly dysfunctional due to hypoxia via mitochondrial stress induced by continuous stimulation (Scharping et al., 2021). We also founded that *GZMB* and *IFNG* are negatively correlated with hypoxia risk score. As a T cell activation marker, the expression of *GZMB* is important for tumor immunity (Russell and Ley, 2002). Moreover, patients with osteosarcoma and a high expression of *GZMB* have a long overall survival time (Yoshida et al., 2019). The expression of Neuronal pentraxin (*NPTX2*) is negatively correlated with the expression of *GZMB* and *IFNG*, Knockdown of *NPTX2* in osteosarcoma cells inhibited tumor growth and increased tumor cell apoptosis (Yang et al., 2021). Besides, some of the immune-related factors associated with tumor invasion and metastasis such as *HMGB1* and Toll-like receptor 4 (*TLR4*) play a considerable role in rebuilding TIM and promoting the development of pyroptosis that could also be upregulated by hypoxia (Liu et al., 2015; Wu et al., 2018; Tan et al., 2020; Zha et al., 2020; Yuan et al., 2021). For example, *HMGB1* promotes lung metastasis in triple-negative breast cancer by inducing CD62L^dim^ neutrophil polarization (Wang et al., 2020b). The prognostic role of *TLR4* in Osteosarcoma remain controversial. As we know, *TLR4* recognizes cathepsin K to activate the M2 polarization of tumor-associated macrophages (*TAMs*) through an mTOR-dependent pathway, resulting in tumor metastasis (Li et al., 2019). It has been reported that the activation of *TLR4* prevents progression of osteosarcoma, while some other evidence demonstrated the *TLR4* related pathway could promote the invasion and migration of Osteosarcoma (Wang et al., 2019b; Zhou et al., 2020b; Yahiro et al., 2020). Thus, the role of *TLR4* in Osteosarcoma need further more investigations. Consistent with the above evidence, our study suggests that CD8^+^ T cells and CD4^+^ T memory-activated cells were decreased in patients with high hypoxia risk, indicating an immunosuppressive status. In our study, the expression of *HMGB1* is higher in normal tissues than that in osteosarcoma from TARGET dataset. However, the immunofluorescence study showed high expression of *HMGB1* in osteosarcoma. It has been reported that the expression of *HMGB1* mRNA is higher in moderately and poorly differentiated gastrointestinal adenocarcinoma tissues compared to adjacent pre-cancerous and well-differentiated tissues (Xiang et al., 1997). It reminds that the expression of *HMGB1* may be associated with the level of differentiation. Besides, there was also high degree of heterogeneity within individual tumors or between tumors (Hasegawa et al., 1991; Klangjorhor et al., 2020). These discordances may have resulted in the difference. These results show that our hypoxia risk model distinguishes immune status and immune cell subtypes in different patients and may be helpful in the selection of appropriate treatments. On the other hand, the above evidence indicates that hypoxia is a predominant driver of TIM remodeling, not only downregulating subset of infiltrating immune cells (e.g., CD8^+^ T cells and CD4^+^ T memory activated cells) but further regulating the immune response-related chemokines and their receptors and thus upregulating pyroptosis. Above all, hypoxia may initiate a series of chain reactions that lead to the suppressive immune status.

As a novel form of programmed cell death, pyroptosis has received increasing attention because of its dual regulation of the tumor immune response. In this study, we developed a pyroptosis risk score model based on DEGs related to pyroptosis that predicts prognosis, chemoresistance, and subtype of immune cells. ssGSEA revealed that the low pyroptosis risk score group had a low proportion of CD8^+^ T cells, Th1 cells, Th2 cells, and low activity in some immune pathways, indicating that pyroptosis also contributes to a suppressive microenvironment. Moreover, T helper cells and CD8^+^ T cells are regarded as crucial immune cells in antitumor immune response, and their large infiltration into tumor tissues is often linked with better prognosis (Fridman et al., 2012). The application of chemotherapy has improved the survival outcomes of patients with OS. Therefore, we evaluated the ability of the pyroptosis risk score to predict the sensitivity of conventional chemotherapies (Strauss et al., 2021). As previously reported, high pyroptosis scores were associated with lower chemosensitivity. After a series of analyses, we found that the low pyroptosis risk score was often associated with better clinical outcomes such as survival, chemosensitivity, and immune status, while the pyroptosis signature showed good predictive ability in these aspects. The above results demonstrate that hypoxia can also contribute to poor prognosis and low chemosensitivity by promoting pyroptosis.

In summary, this study has revealed the relationship between hypoxia, pyroptosis, and OS. Hypoxia is closely connected to the development of OS because the HRGs are differentially expressed in healthy and OS tissues, and could promote an immunosuppressive microenvironment by modulating the infiltration of immune cells and immune-related factors. In addition, our HRG and PRG signatures can predict the prognosis and immune landscape status in OS patients, thereby providing novel insights into OS tumorigenesis and a foundation for novel OS detection and treatment strategies.

## Conclusion

In conclusion, we have established and validated a prognostic model based on HRGs in patients with OS that can also evaluate the characteristics of the tumor immune landscape. Moreover, another pyroptosis risk-score model that includes 14 PRGs provides independent prognostic and chemosensitivity capabilities. Through the complicated connections between hypoxia and pyroptosis, this study has demonstrated that hypoxia could alter poor prognosis and chemoresistance by affecting TIM remodeling and the occurrence of pyroptosis.

## Data Availability

The datasets presented in this study can be found in online repositories. The names of the repository/repositories and accession number(s) can be found in the article/Supplementary Material.

## Abbreviations

DEGs, Differential expressed genes; DCN, Decorin; GEO, Gene Expression Omnibus; GO, gene ontology; GTEx, genotype-tissue expression database; GSEA, gene set enrichment analyses; GZMB, Granzyme B; HRGs, Hypoxia-related genes; HRG, hypoxia-related genes; HOMX1, Heme oxygenase-1; IFNG, Interferon Gamma; KEGG, kyoto encyclopedia of genes and genomes; LOX, lysyl oxidase; OS, Osteosarcoma; PRGs, pyroptosis related genes; PPI, The protein-protein interaction; PDK1, pyruvate dehydrogenase kinase 1; TARGET, Therapeutically Applicable Research to Generate Effective Treatments; TIM, tumor immune microenvironment; ssGSEA, Single sample GSEA; PBS, phosphate buffer solution; TLR4, Toll-like receptor 4; TAMs, tumor-associated macrophages; NPTX2, Neuronal pentraxin.
