# Supplementary figures and images for: The hypoxia-related signature predicts prognosis, pyroptosis and drug sensitivity of osteosarcoma
Source: Front Cell Dev Biol. 2022 Sep 20;10:814722. doi: 10.3389/fcell.2022.814722 (PMC9532009; doi:10.3389/fcell.2022.814722)

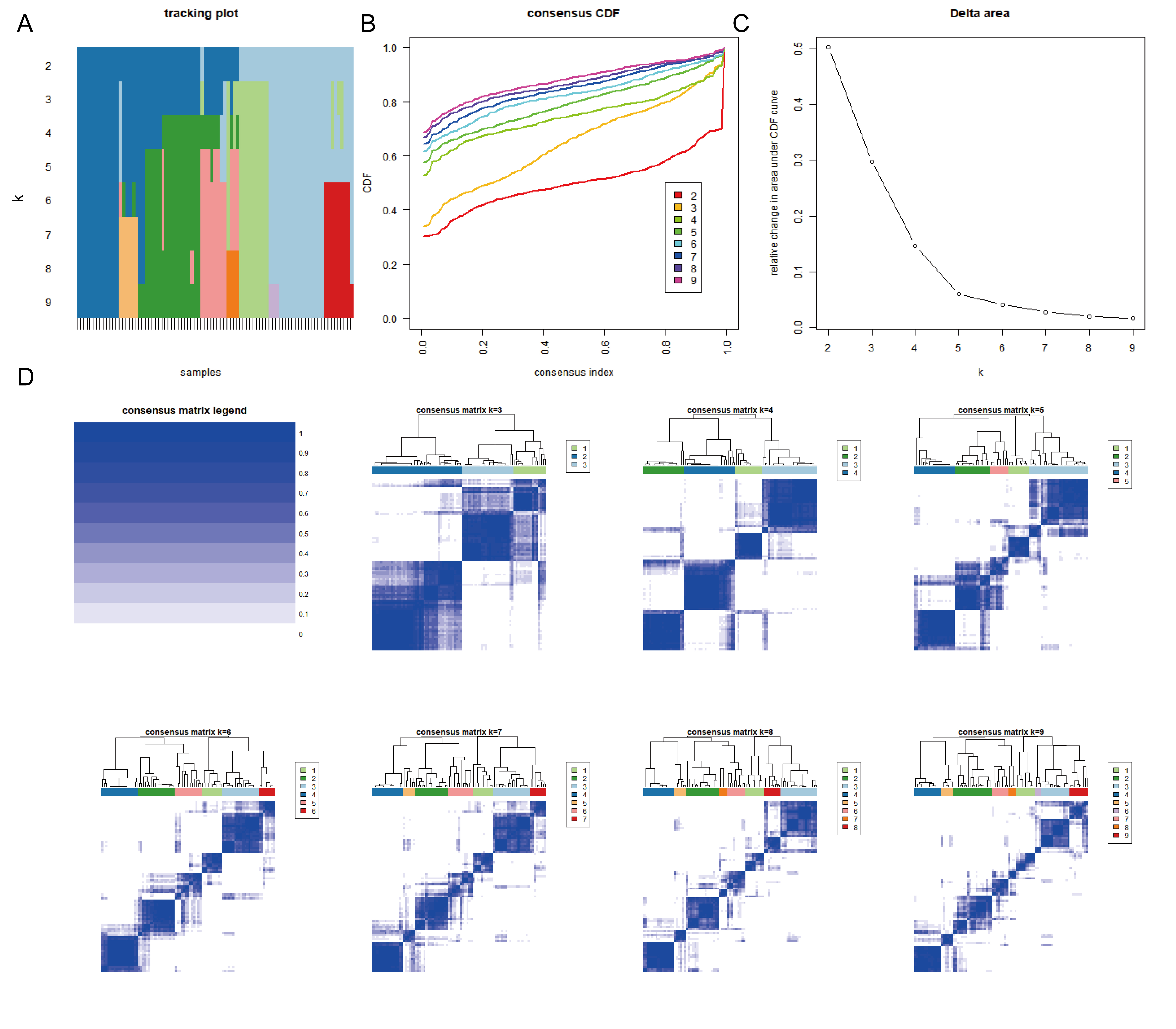

Supplement: Supplementary file 1 [file Image3.TIFF]

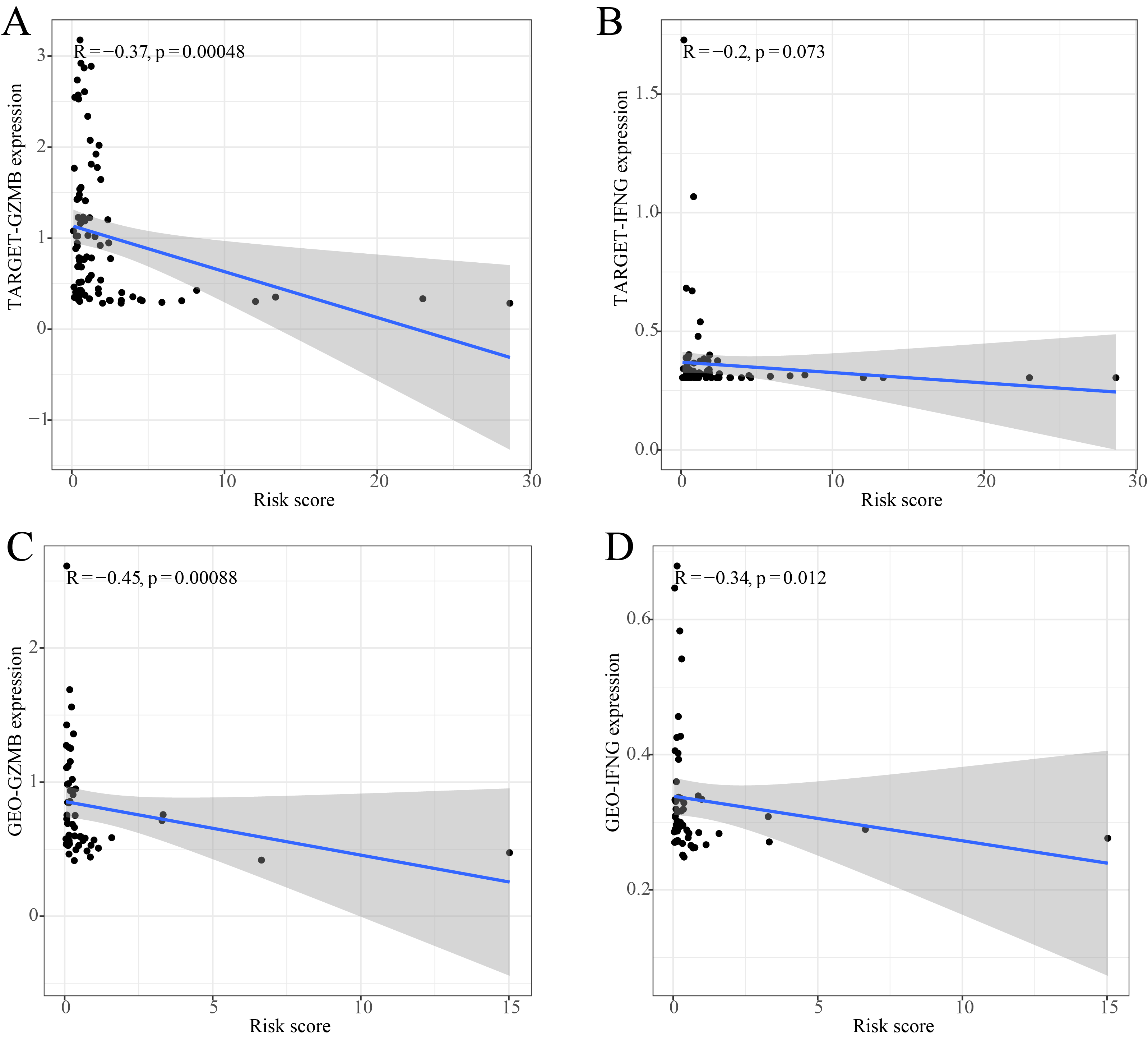

Supplement: Supplementary file 3 [file Image2.TIF]

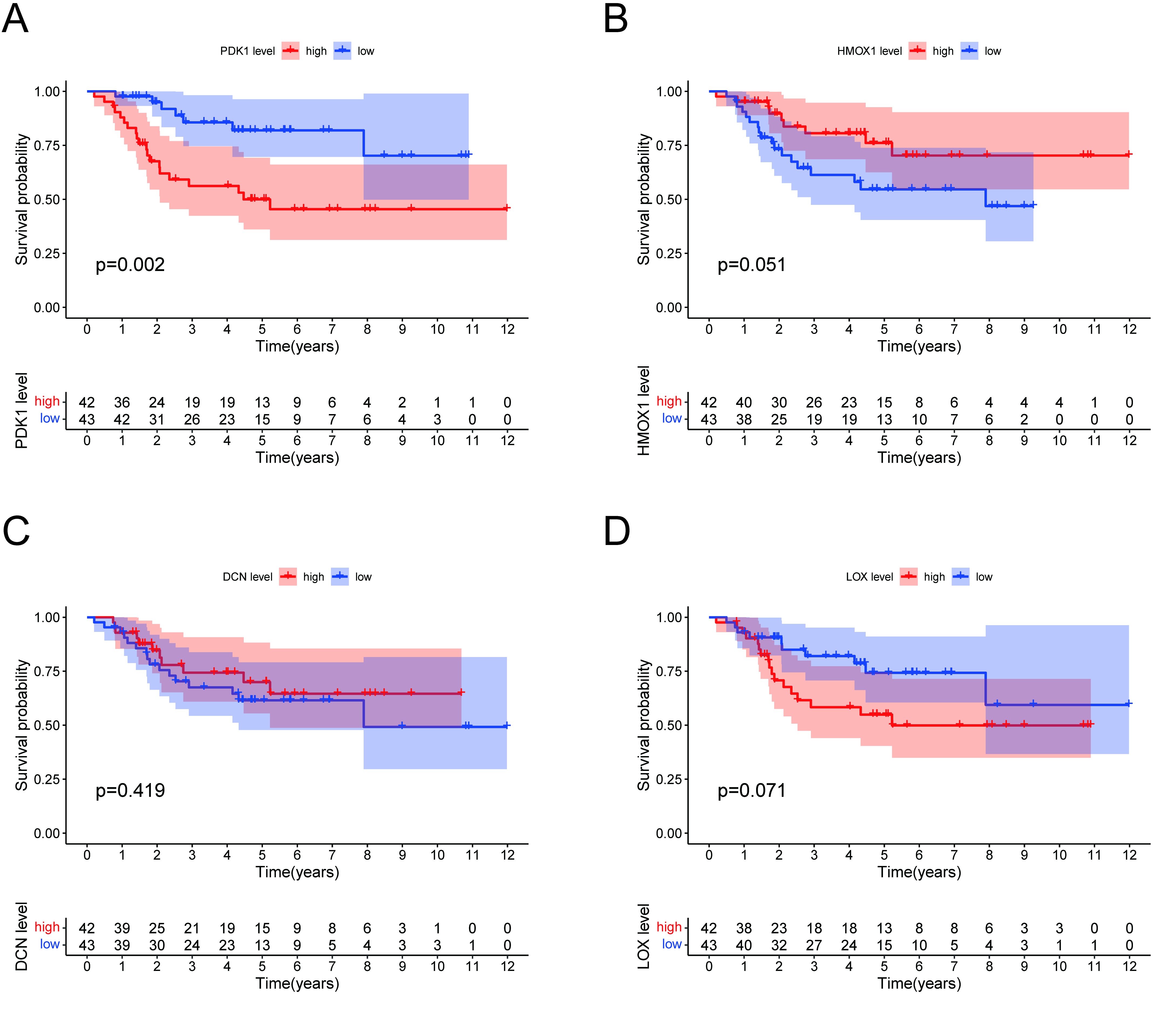

Supplement: Supplementary file 4 [file Image1.TIF]

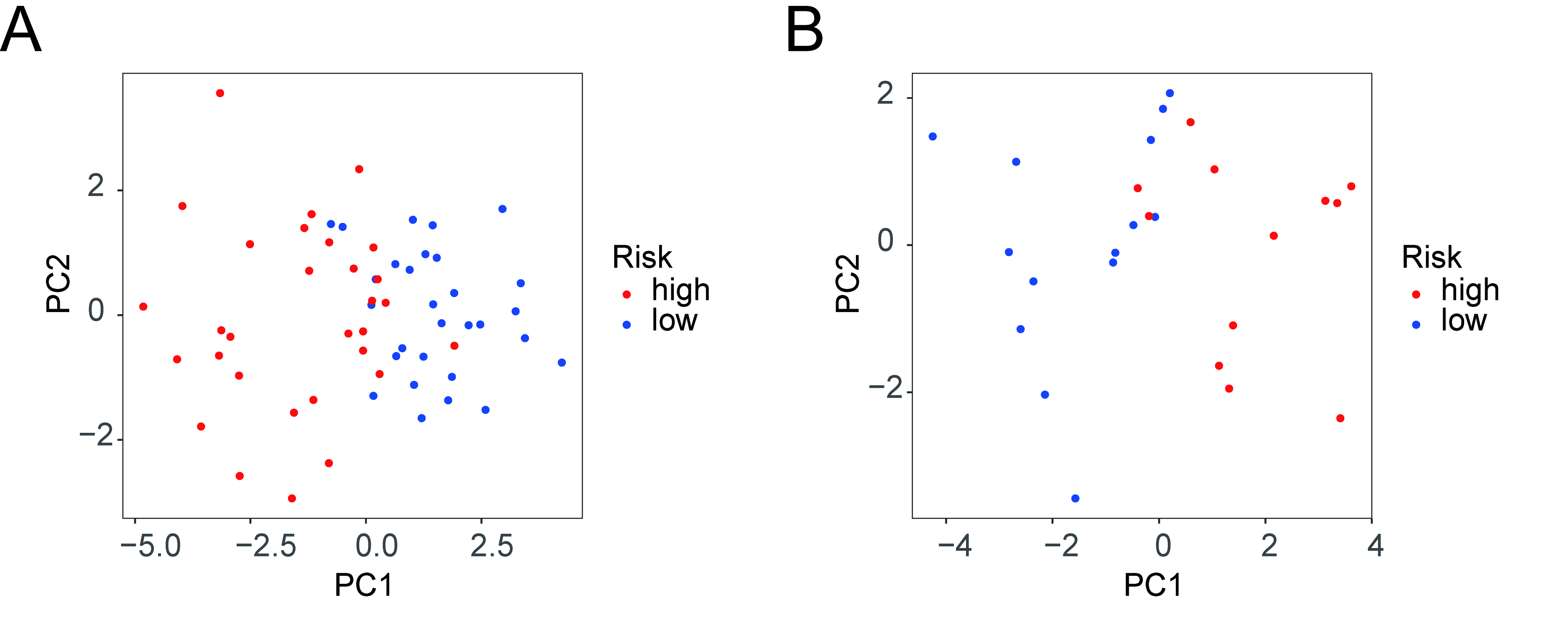

Supplement: Supplementary file 6 [file Image5.TIF]

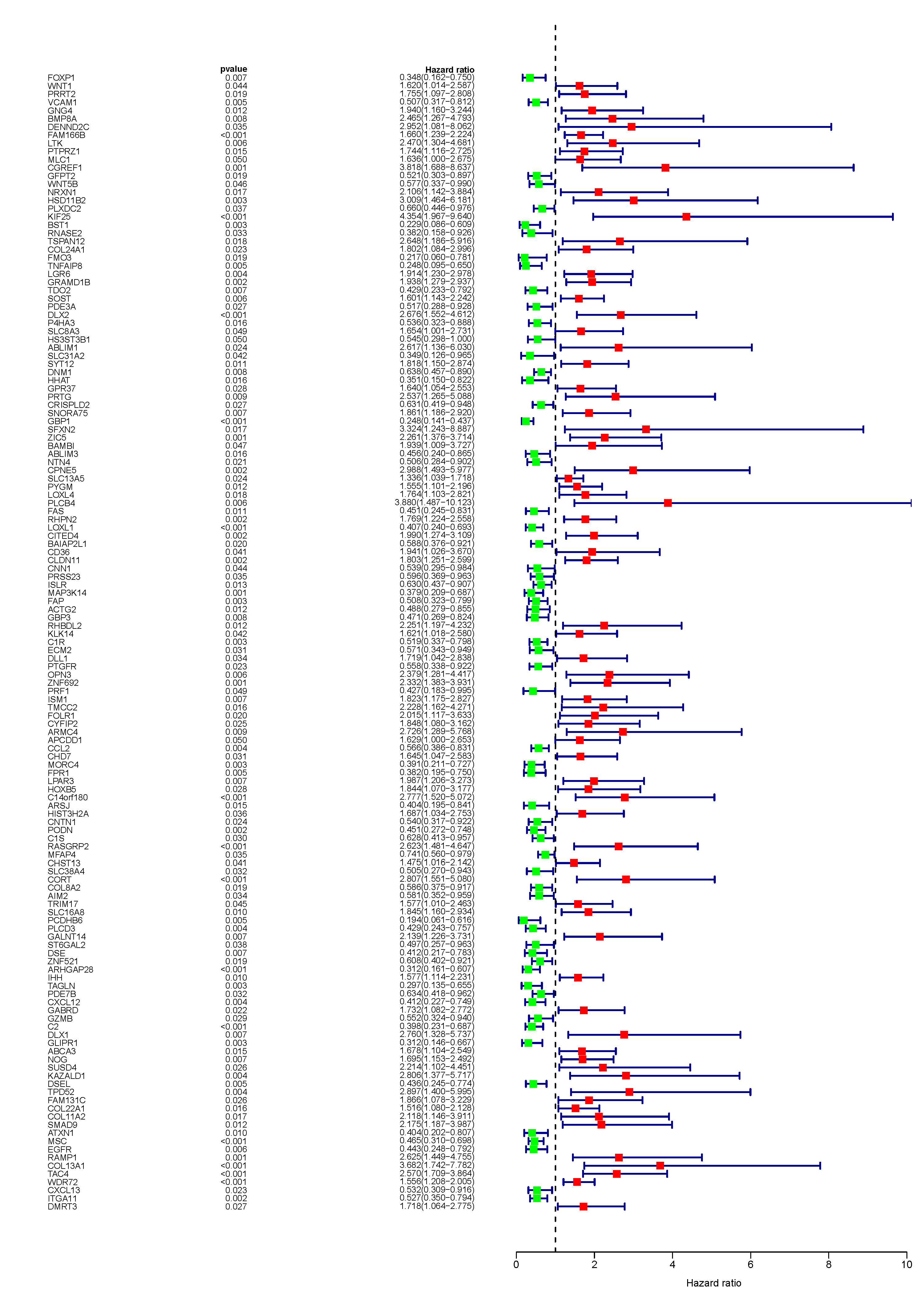

Supplement: Supplementary file 7 [file Image4.TIFF]
