# Supplementary material for: The hypoxia-related signature predicts prognosis, pyroptosis and drug sensitivity of osteosarcoma
Source: Front Cell Dev Biol. 2022 Sep 20;10:814722. doi: 10.3389/fcell.2022.814722 (PMC9532009; doi:10.3389/fcell.2022.814722)
Supplement: Supplementary file 2 [file Table1.docx]

| **Table S1. Primer sequence of siRNA** | | |
| --- | --- | --- |
| **sequence of siRNA** | **SS Sequence** | **AS Sequence** |
| HMGB1-SiRNA1 | GGCCCGUUAUGAAAGAGAATT | UUCUCUUUCAUAACGGGCCTT |
| HMGB1-SiRNA2 | GGAGAGAUGUGGAAUAACATT | UGUUAUUCCACAUCUCUCCTT |
| TLR4-SiRNA1 | GGCUGUGGAGACAAAUCUATT | UAGAUUUGUCUCCACAGCCTT |
| TLR4-SiRNA2 | GGCUCACAAUCUUAUCCAATT | UUGGAUAAGAUUGUGAGCCTT |
| CCL28-SiRNA1 | GAGUCUACAGAUAAAUCUATT | UAGAUUUAUCUGUAGACUCTT |
| CCL28-SiRNA2 | CUGUCAUCCUUCAUGUCAATT | UUGACAUGAAGGAUGACAGTT |
| KLF2-SiRNA1 | GCACCGACGACGACCUCAATT | UUGAGGUCGUCGUCGGUGCTT |
| KLF2-SiRNA2 | GCGGCAAGACCUACACCAATT | UUGGUGUAGGUCUUGCCGCTT |
| TNFSF18-SiRNA1 | GGCUAAGUUUGGACCAUUATT | UAAUGGUCCAAACUUAGCCTT |
| TNFSF18-SiRNA2 | GGUUGGAUCUGCAGAGAUATT | UAUCUCUGCAGAUCCAACCTT |
