# Supplementary material for: The hypoxia-related signature predicts prognosis, pyroptosis and drug sensitivity of osteosarcoma
Source: Front Cell Dev Biol. 2022 Sep 20;10:814722. doi: 10.3389/fcell.2022.814722 (PMC9532009; doi:10.3389/fcell.2022.814722)
Supplement: Supplementary file 5 [file Table2.docx]

| **Table S2. Genes used for establishment of signature and coefficient values** | | | | | | |
| --- | --- | --- | --- | --- | --- | --- |
| **Gene** | **Coefficient** |  |  |  |  |  |
| HSD11B2 | 0.496305 |  |  |  |  |  |
| KIF25 | 0.115642 |  |  |  |  |  |
| BST1 | -0.0646 |  |  |  |  |  |
| SNORA75 | 0.03272 |  |  |  |  |  |
| GBP1 | -0.69757 |  |  |  |  |  |
| CLDN11 | 0.073825 |  |  |  |  |  |
| ZNF692 | 0.045101 |  |  |  |  |  |
| ARMC4 | 0.012827 |  |  |  |  |  |
| FPR1 | -0.13367 |  |  |  |  |  |
| PCDHB6 | -0.16551 |  |  |  |  |  |
| IHH | 0.027013 |  |  |  |  |  |
| TPD52 | 0.099566 |  |  |  |  |  |
| RAMP1 | 0.114857 |  |  |  |  |  |
| TAC4 | 0.300302 |  |  |  |  |  |
